# Artificial intelligence at work: The problem of managerial control from call centers to transport platforms

**DOI:** 10.3389/frai.2022.888817

**Published:** 2022-08-25

**Authors:** Jamie Woodcock

**Affiliations:** Department of People and Organisations, The Open University, Milton Keynes, United Kingdom

**Keywords:** artificial intelligence, algorithmic management, labor process, call centers, platform work, gig economy

## Abstract

There has been much recent research on the topic of artificial intelligence at work, which is increasingly featuring in more types of work and across the labor process. Much research takes the application of artificial intelligence, in its various forms, as a break from the previous methods of organizing work. Less is known about how these applications of artificial intelligence build upon previous forms of managerial control or are adapted in practice. This paper aims to situate the use of artificial intelligence by management within a longer history of control at work. In doing so, it seeks to draw out the novelty of the technology, while also critically appraising the impact of artificial intelligence as a managerial tool. The aim is to understand the contest at work over the introduction of these tools, taking call centers and transport platforms as case studies. Call centers are important because they have been a site of struggle over previous forms of electronic surveillance and computation control, providing important lessons for how artificial intelligence is, or may, be used in practice. In particular, this paper will draw out moments and tactics in algorithmic management has been challenged at work, using this as a discussion point for considering the possible future of artificial intelligence at work.

## Introduction

Artificial intelligence is a broad category of digital technologies that involve intelligence demonstrated by computers and machines. The definition of intelligence used here is broad, covering examples of search engines, recommendations of what to watch next on streaming services, all the way up to the artificial general intelligence of science fiction. As Cook (2018) has argued, “many people are misrepresenting AI in order to make it appear more intelligent than it is.” In part, this is due to the aggressive marketing of new technology to both investors and consumers. Similarly, Taylor (2018) has coined the term “fauxtomation”, explaining how “automated processes are often far less impressive than the puffery and propaganda surrounding them imply—and sometimes they are nowhere to be seen.”

There has been much discussion in the context of platform work on the role of artificial intelligence and algorithms (Srnicek, 2017). More widely, there has been research interest in artificial intelligence, robotics, and other advanced technologies for use in the workplace, with one paper finding 13,136 potentially relevant studies (Vrontis et al., 2022). Following the increasing popularity of research on the topic in general (Pasquale, 2015; Kitchin, 2017; O'Neil, 2017; Turow, 2017; Eubanks, 2019), research on Uber has often focused on algorithm management (Lee et al., 2015; Rosenblat and Stark, 2016; Scholz, 2017; Rosenblat, 2018). For some in the literature, this is seen as a new attempt by management to overcome worker resistance (Veen et al., 2019; Mahnkopf, 2020), while others have drawn attention to the experience of workers struggling against these new techniques (Waters and Woodcock, 2017; Fear, 2018; Briziarelli, 2019; Cant, 2019; Gent, 2019; Leonardi et al., 2019; Cant and Mogno, 2020; Tassinari and Maccarrone, 2020).

The aim of this article is to engage with the topic of artificial intelligence at work over a longer history of supervision and control at work, drawing on empirical and conceptual research. It starts by considering this history and the lessons that can be taken from call center work. The article then moves on to discuss labor process theory and systems of control in factories, call centers, and transport platforms. This provides the theoretical framing for the argument that is explored through the case studies and the exploration of algorithmic management in practice. The article ends by considering how this can shape our understanding of the strengths and limits of artificial intelligence in general, and specifically algorithmic management, at work. The intention is to develop an argument about the significance of artificial intelligence, not as a general technology, but as a form of surveillance and control in the workplace. This is important for both its implementation, but also for understanding struggles against its use.

## Approach

This is a conceptual paper that draws on existing research on call centers and platforms. The author has conducted substantial ethnographic fieldwork in call centers (Woodcock, 2017) as well as with the transport platforms (Woodcock and Graham, 2019; Woodcock, 2021) that contribute empirical data toward the argument in this article. The approach taken here is an attempt to draw these findings, as well as those from other research in the field, into a conceptual argument about the role of artificial intelligence at work. This involved the synthesizing of findings from traditional factory settings, call centers, and transport platforms, to conceptualize the role of systems of control within the labor process. This draws primarily from the literature in labor process theory, combined with critical research on algorithms and power more widely.

## Call centers, surveillance, and control

There is a long history of control at work. From the moment that bosses started buying workers' labor-power, there have been successive attempts to watch and control what workers are doing at work. Taylor (1967) identified this as the fear of “soldiering” in his theory of scientific management, the belief that workers would deliberately work slower than they could. This managerial fear was not limited to Taylor or Taylorism and is present throughout many kinds of work.

Before the emergence of platform work, call centers were a focus for debates on technological surveillance and control. These debates are useful to revisit in the context of artificial intelligence, particularly as many of the forms of electronic surveillance and outsourcing developed in call centers laid the basis for the technical organization of platform work. Call centers were an important focus of debates on technology, control, and resistance (Woodcock, 2017). The technical arrangements of the labor process made call center work particularly susceptible to early attempts at electronic surveillance and control. As the phone systems were integrated with computers, this provided the possibility to use new technologies in a way that would have been harder to achieve in other forms of low paid work.

Call centers provide an important early example of work that could be digitally legible (see Woodcock and Graham, 2019) that allows it to be measured through discrete data points. Through the integration of telephones and computers, facilitated by the development of automatic call distributors, the modern call center was established. This took away the control from call center workers, automating the process of dialing and speeding up the work. It created the experience of an “assembly line in the head” for call center workers (Taylor and Bain, 1999, p. 103). The new technology also provides a way to electronically supervise the labor process. The computerisation of the process involved developing the capacity to measure each part of the labor process: how many calls made, successful sales, length of calls, time between calls, breaks, and other metrics. Given work in a call center requires a range of clear quantitative indicators, these could now be collected automatically. As I found in a call center in the UK, these “quantitative variables are context free; not something that can be debated, considered instead as the evidence base for rewards or discipline” (Woodcock, 2017). The scale of this data collection is impressive: it “allows an unprecedented level of surveillance; every call encounter is permanent, every mistake could be punishable in the future. It operates like the ability to recall every commodity produced on an assembly line and to be able to retrospectively judge the quality of its production” (Woodcock, 2017).

There are many studies of call centers that have confirmed similar findings (Taylor and Bain, 1999; Bain et al., 2002; Kolinko, 2002; Mulholland, 2002). However, there is also evidence that call centers had aggressive management techniques that preceded the development of these new technological methods. For example, as an interviewee explained:

There were all sorts of rules right. I mean for instances hanging coats on the back of your chair was banned, little things like that. Constantly listing things that people couldn't do. I've seen people being chased into toilets because they have their phones on them and stuff like that! All these things you can do with or without the computers (quoted in Woodcock, 2017).

It is therefore important to remember that new technological forms of surveillance and control are developed and implemented within the existing social relations of the workplace—even if they then go on to transform them further.

There is a broad existing literature on call centers that has produced detailed understandings of “work organization, surveillance, managerial control strategies and other central concerns of labor process analysis” (Ellis and Taylor, 2006, p. 2). The key debates within the literature centers around the extent and implications of new technological forms of control. On one side of the debate were academics who argued that call centers were becoming organized like an “electronic panopticon.” For example, Fernie and Metcalf (1997, p. 3) claims that the “possibilities for monitoring behavior and measuring output are amazing to behold—the ‘tyranny of the assembly line' is but a Sunday school picnic compared with the control that management can exercise in computer telephony.” This notion of an electronic panopticon—which draws heavily on both Foucault (1991) and Bentham (1995) and the architectural model of a prison—has similarities with some of the contemporary debates on algorithmic management. However, on the other side of the debates, McKinlay and Taylor (1998, p. 75) argued that the comparison fails to take into account that “the factory and the office are neither prison nor asylum, their social architectures never those of the total institution.” Indeed, as Taylor and Bain (1999, p. 103) argue, the “dynamic process of capital accumulation” that takes place in the workplace means that Foucauldian approaches drawing on the panopticon analogy “understates both the voluntary dimension of labor and the managerial need to elicit commitment from workers.” This has important implications for theorizing work, particularly that it can “disavow the possibilities for collective organization and resistance” (Taylor and Bain, 1999, p. 103).

These debates can be revisited in a more productive way today, particularly tracing the development from factory supervision, call centers, and then to contemporary platforms (Woodcock, 2020). The claims about the novelty or scope of technological changes today can be reinterpreted through these older debates, providing important theoretical grounding, as well as reminder about the continuing dynamics of work. For example, Taylor and Bain (1999) argument reminds us that technological methods of control cannot solve the problems of management. In the call center, vast quantities of data are collected, but human supervisors are still required to interpret the data and act upon any insights. There are 1-2-1 meetings, coaching, training, and “buzz sessions” that attempt to elicit motivation from workers on the call center floor (Woodcock, 2017). In the context of call center work, there is “no electronic system can summon an agent to a coaching session, nor highlight the deficiencies of their dialogue with the customer.” Instead, as Taylor and Bain (1999, p. 108-109) continue, call centers “rely on a combination of technologically driven measurements and human supervisors”, which nevertheless “represents an unprecedented level of attempted control which must be considered a novel departure.”

## From call centers to platforms

In order to apply these lessons to our understanding of artificial intelligence at work, it is therefore necessary to return to the concerns of labor process theory (both in the call center and more widely) to understand the implications of these new management techniques. A “common feature of all digital labor platforms is that they offer tools to bring together the supply of, and demand for, labor” (Graham and Woodcock, 2018). Regardless of whether the legal categorization is employment or self-employment (De Stefano and Aloisi, 2019), these platforms involve work. The labor process is coordinated *via* a digital platform and in the case of transport platforms, often involves a smartphone and GPS. The rapid growth of food delivery and private hire driving platforms has been facilitated by the digital legibility of the labor process, involving discrete data points of start and end journeys.

The concerns of labor process theory involve understanding what happens in the workplaces after the purchase of workers labor-power by capital. This involves the “indeterminacy of the labor process” that requires managing in practice. For example, Edwards (1979, p. 12) argues that:

conflict exists because the interests of worker and those of employers collide… control is rendered problematic because unlike the other commodities involved in production, labor power is always embodied in people, who have their own interests and needs and who retain their power to resist being treated like a commodity.

The act of mediating these relationships on a platform does not remove the different interests or make the distributed workplace any less of a “contested terrain.” Edwards (1979, p. 18) provides a three-part framework for understanding the “system of control” in the workplace. The first is “direction”, or the ways in which the tasks that workers have to do are specified. The second is “evaluation”, or how the employer supervises and assesses the workers performance. The third is “discipline”, or what methods are used “to elicit cooperation and enforce compliance with the capitalist's direction of the labor process.”

As Table 1 illustrates, systems of control can be broken down into the three aspects to develop a more specific understanding of how control is operating in practice. The first thing to note is that elements of automation are present throughout each example. Automation is not the preserve of algorithms, nor is it a simple binary. From the moment that workers started to use tools and machines at work, parts of the labor process began to become automated. It is rare that tasks are ever completely automated, instead the element of human labor becomes decreased—sometimes drastically. For example, as factories have developed since the industrial revolution, the individual productivity of workers has increased by huge amounts. Yet there are still workers in factories. Even in so-called “lights out” factories, workers are required for setting up manufacturing tombstones, quality assurance checks, and the repair and maintenance of machinery.

**Table 1:** Systems of control.

	**Factory**	**Call center**	**Transport platform**
Direction	Taylorist separation of conception and execution of work, workers given specific instructions. Assembly line automatically sets central pace	Separation of conception and execution of work with scripting. Automatic dialing of calls increases pace	Separation of conception and execution of overall work on platform. Workers receive direction through smartphone, but can have discretion with route choices
Evaluation	Supervisors assess the labor process on the factory floor, quality assurance of outputs	Quantitative metrics from electronic supervision, qualitative evaluation by supervisors	Automated evaluation of the labor process with quantitative metrics. Customer evaluation in some cases
Discipline	Supervisors encourage performance, bonuses can be used to increase output. Sanctions for poor performance	Supervisors encourage performance, bonuses used to increase output. Sanctions for missing targets	Use of bonuses to encourage engagement at peak times. Automated interventions based on automated evaluation (“deactivations”)

Table 1 shows how the traditional operation of factories involves aspects of automation, but relies upon a layer of supervisors who monitor, assess, and intervene in the labor process. It builds on the classical Taylorist division of labor and the separation of the conception of tasks from their execution. This involves management attempting to take control away from the workplace, directing workers to complete tasks in specific ways and within set times. It is also worth noting that before the theory was applied to call centers, Foucault (1991, p. 174) wrote about supervision in a factory context. He argued that it involved:

an intense, continuous supervision; it ran right through the labor process; it did not bear – or not only—on production… It became a special function, which had nevertheless to form an integral part of the production process, to run parallel to it throughout its entire length. A specialized personnel became indispensable, constantly present and distinct from workers.

The obsession with measurement and supervision that begins in the factory becomes applied to an increasing range of work.

Call centers represent a significant development from this model of control. The separation of conception and execution is developed through a form of computational Taylorism and scripting of the phone calls that workers made (Woodcock, 2017). The integration of computers and telephones the collection and digital storage of a range of quantitative metrics, as well as recordings of calls. However, this data requires supervisors to interpret and intervene in order for it to be productive in the workplace. This is not a straightforward process in call centers, with many having high levels of turnover. Instead, disciplinary actions are combined with attempts to motivate workers and the use of monetary bonuses. The role of supervisors develops from the factory floor, particularly in relation to handling abstract data on the labor process, but remains a key interface between workers and capital.

The shift to transport platforms involves the development of control across each of the three component parts. However, one of the key differences is that there is no longer a formal employment arrangement. This means that many of the tools that are available in other kinds of work cannot be used, less the platform risks workers being reclassified away from self-employment (Woodcock and Cant, 2021). With transport platforms there are clear start and end points, with points of contact with either other workers or customers. The work is suitable for metrics in a way that would be harder for other forms of low paid work like cleaning or care. The majority of the metrics are quantitative (how long did the task take) rather than qualitative (how well was the task completed). Similarly, this form of work organization has developed alongside a specific form of contractual relationship: independent contractor or self-employment status.

However, across each case, the aim of the process is to elicit motivation for workers to complete tasks in the labor process. In the factory and the call center, this means trying to overcome the indeterminacy of the labor process, ensuring that capital gets the full value (or, at least, as much as it can) from the purchase of workers labor-power. The problem with this, as Thompson (1983, p. 123) reminds us, is that “complications arise when attempts are made to specify how control is acquired and maintained.” Workers want to have energy left after a shift ends—and often there is no benefit to working harder. The widespread use of bonuses can be seen as one solution to this problem, as well as the development of increasing complex methods of supervision and surveillance. Control can mean, in “an absolute sense, to identify those ‘in control', and in a relative sense, to signify the degree of power people have to direct work” (Thompson, 1983, p. 124). That degree of power can shift with the use of new techniques and technologies. Indeed as Goodrich (1975) notes, there is always a dynamic “frontier of control” in the workplace that pushes back and forth between the different interests of workers and capital.

## Artificial intelligence as technology of workplace control

To talk about artificial intelligence in general terms in the workplace is not meaningful. It involves, as noted early, many forms of simple and more complex artificial intelligence that are proliferating throughout work. At the core, algorithms involve “sets of defined steps structured to process instructions/data to produce an output” (Kitchin, 2017, p. 14). In more complex iterations, this can involves very large or rapid processes, meaning the operation can be obscured as if they operate like a “black box” (Pasquale, 2015). In many cases, algorithms do not shift the frontier of control between capital and labor in any substantial way. For example, autocomplete options in emails are not likely to effect widespread changes in the balance of power in the workplace. However, automated decision making over shift bookings can have a tangible impact on the experience of work.

The development of platform work has provided an important “laboratory for capital” (Cant, 2019), experimenting with new uses for artificial intelligence and automation in the organization of delivery work. However, it has also involved the specific contractual relationships noted above. Instead of entering into formal employment contracts with workers, platforms instead seek to engage workers as self-employed contractors. This misclassification of workers means that platforms can evade the protections and liabilities they would other have to take on with conventional employment models. This model has facilitated the rapid expansion of platforms, particularly in transportation, but it also prevents platforms from acting like employers in some instances. Given the challenges to employment status in many jurisdictions, some platforms have responded by limiting training and communication to ensure they will not fail employment status tests (see, with Deliveroo, Woodcock and Cant, 2021).

Without the traditional forms of workplace control, platforms rely upon algorithmic management to manage a dispersed workforce. Due to the employment status issues, physical supervision is no longer an option, removing interventions like calling workers in for disciplinary meetings or performance improvement meetings, while limiting communication across the platform. One of the basic functions of supervision—telling workers to work harder—is therefore more complicated to achieve in practice. Instead, platforms can use Service Level Agreements and other contractual tools, setting targets in the hope that workers will try to meet them. Instead of direction supervision, this involves a wider set of practices that seek to “seduce, coerce, discipline, regulate and control: to guide and reshape how people… interact with and pass through various systems” (Kitchin, 2017, p. 19). One example of this is the bonus structure, including “boosts” for deliveries during busy periods or adverse weather conditions. This incentivizes workers to log onto the platform, rather than requiring it through strict scheduling. Other strategies are more direct. For example, the use of “deactivation” or firing workers who do not meet performance targets—or some other algorithmically determined reason. The strengths and weaknesses of this approach are considered in Table 2.

**Table 2:** Algorithmic systems of control.

	**Transport platform**	**Strengths**	**Weaknesses**
Direction	Separation of conception and execution of overall work on platform. Workers receive direction through smartphone, but can have discretion with route choices	In a straightforward task with clear start and finish this is an effective way of distributing instructions	If there are problems during the labor process, there are few options available to workers to negotiate the process. It is not effective with complex tasks or those without clear start or end points
Evaluation	Automated evaluation of the labor process with quantitative metrics. Customer evaluation in some cases	The labor process creates data on locations and timings that is straightforward to track. Customer feedback can be quickly collected	It is difficult to accurately evaluate qualitative aspects of the labor process
Discipline	Use of bonuses to encourage engagement at peak times. Automated interventions based on automated evaluation (“deactivations”)	Bonuses can encourage workers to work. Threat of “deactivations” can play a disciplinary function	Bonuses increase the cost of labor-power and may not achieve aim of the labor process. Workers can find ways to game the system. There are no intermediate disciplinary actions before “deactivation”

As can be seen in Table 2, algorithmic systems of control at work have both strengths and weaknesses. In the case of food delivery platforms, this has involved the removal of a supervisors or managerial layer from the work, instead relying upon automated decision-making processes. This has proven to be a successful model for organizing work—at least for the majority of the time. However, this “platform management model” is contested by workers in practice (Moore and Joyce, 2019). The weaknesses of the approach can be seen when workers actively resist platform control, particularly during strike action. It is during these moments that the lack of managerial intervention (disciplinary or otherwise) shows that there are two kinds of precariousness at Deliveroo, both for the workers involved, but also for the management of the platform (Woodcock, 2020).

Building from the arguments about the “electronic panopticon” (Fernie and Metcalf, 1997), the metaphor can also be used to make sense of algorithmic management (Woodcock, 2020). Unlike the physical architecture of the prison, it is possible to see how the dynamics of the panopticon operate on a platform like Deliveroo. The work involves discrete tasks that increase in frequency during peak times, particularly lunch and dinner. The role of supervision, algorithmic or otherwise, involves trying to ensure that the purchased labor-power is used most effectively. As Foucault (1991, p. 150) noted in the context of the factory: “to assure the quality of the time used: constant supervision, the pressure of supervisors, the elimination of anything that might disturb or distract; it is a question of constituting a totally useful time.” While this has developed significantly from hiring human supervisors to prowl the workplace, it still involves finding ways to discipline time, as “time measured and paid must also be a time without impurities or defects; a time of good quality, throughout which the body is constantly applied to its exercise” (Foucault, 1991, p. 150).

This point about time is important, as it underlined the original arguments for the panopticon. Bentham (1995, p. 80) argued that the panopticon could find uses beyond the prison: “whatever be the manufacture, the utility of the principle is obvious and incontestable, in all cases where the workmen are paid according to their time.” The panopticon was therefore also considered as a potential solution to the problem of the indeterminacy of labor power. Bentham continued to argue that the panopticon could be combined with a piece rate payment scheme, as “there the interest which the workman has in the value of his work supersedes the use of coercion, and of every expedient calculated to give force to it” (Bentham, 1995, p. 80). Foucault, of course, took this further, arguing that the panopticon as an “architectural apparatus should be a machine for creating and sustaining a power relation independent of the person who exercises it; in short, that the inmates should be caught up in a power situation of which they are themselves the bearers” (Foucault, 1991, p. 201).

In the context of the call center, this meant the constant threat of supervisors listening in to calls—as well as being able to recall recordings of all previous calls that had been made. Clearly, no supervisor could be listening to all calls taking place at one time in the call center, but it created the sense that they could be. This experience led to Fernie and Metcalf (1997, p. 3) applying the metaphor of the “electronic panopticon”, as discussed above. In many call centers, this is combined with bonus structures, but rarely with payment that is entirely piece rate.

With platform work, the attention is usually on the technology, software, or algorithmic management. These are the “new” features of the work that have gathered substantial attention. Indeed, Foucault (1991, p. 173) discusses how:

the perfect disciplinary apparatus would make it possible for a single gaze to see everything constantly. A central point would be both the source of light illuminating everything, and a locus of convergence for everything that must be known: a perfect eye that nothing would escape and a center toward which all gazes would be turned.

Given the claims made about the potential of algorithmic management, it is easy to see how the automation of these processes looks increasingly like the metaphor of the panopticon. Recent research has used more general terms for the role of algorithms at platforms like Deliveroo. For example, Muldoon and Raekstad (2022) use the concept of “algorithmic domination” to refer to the “dominating effects of algorithms used as tools of worker control.” They argue that “bosses can employ systems of algorithmic domination to control a more flexible labor force.”

There is a risk of considering algorithmic management as a general solution to the problem of controlling the labor process. Much less attention is paid to the fact that much of this work is organized around piece rate payment. The first struggle at Deliveroo in London was organized in response to the platform moving away from payment per hour to only payment by drop (Waters and Woodcock, 2017). Muldoon and Raekstad (2022) consider this in terms of “dynamic pricing”, but the focus quickly returns to the role of algorithms. There is a long history of piece rates being used in many industries, which can provide a challenge, but have definitely not prevented workers collectively organizing.

The while there are a range of practices that algorithmic control can entail, as noted earlier by Kitchin (2017, p. 19), it is also worth considering the role of “seduction” in more detail. Foucault identified the “form of power which makes individual subjects”, both “a form of power which subjugates and makes subject to” (Foucault, 1982, p. 781). This implies a level of consent, albeit produce through the seduction of algorithmic practices, in the labor process. There are similarities here with the argument of “manufacturing consent” (Burawoy, 1979). While this is secondary to the processes unfolding, it remains a consistently present feature of platform work, often seen in the subjectivity that develops around freedom and flexibility. Algorithmic control, therefore, builds on a relation of power developed between platform and worker. In a Foucauldian sense:

it incites, it seduces, it makes easier or more difficult, in the extreme it constrains or forbids absolutely. it is nevertheless always a way of acting upon an acting subject or acting subjects by virtue of their acting or being capable of action. A set of actions upon other actions (Foucault, 1982, p. 789).

This can be seen across Tables 1, 2 with the use of different actions, from the direction, evaluation, and discipline, now transformed away from the direct managerial prerogative of a conventional workplaces through platform technology.

The general surveillance of algorithmic management represents something new, but it does not necessarily mean that workers are now dominated by algorithms. Platforms use technologies that subject workers to new forms of surveillance and attempted control. However, the Foucauldian argument sees workers “become the principle of” their “own subjection” (Foucault, 1991, p. 203). This is the risk of talking about control—or indeed domination—in general terms. Foucault (1991, p. 174) recognized that “the disciplinary gaze did, in fact, need relays… it had to be broken down into smaller elements, but in order to increase its productive function: specify the surveillance and make it functional.” In the call center I studied, workers found ways to oppose surveillance and make it less functional. Mulholland (2004, p. 711) notes that general accounts claim that “management is triumphant, and it is suggested that discipline has replaced conflict, when seductive discourses make workers the captives of organizational values.” The workplace is not a prison and involves different social relations (McKinlay and Taylor, 1998). This is what makes call centers an interesting example, that the innovations of capital at the time represented “an unprecedented level of attempted control” (Taylor and Bain, 1999, p. 109). Due to the different interests in the labor process, management cannot achieve totalising aims, because “control mechanisms embodied significant levels of managerial coercion and therefore attracted varying levels of resistance” (van den Broek, 2004).

Algorithmic management takes this at least one step further than the call center. Instead of the physical supervision at the center of the prison, instead there is an automated collection of data that runs throughout the entire labor process. As I found in my research with Deliveroo riders, the algorithmic process goes beyond measurement, but rely upon illusions of control and freedom. The threat of algorithmic management is not total and has many gaps and issues in practice. Workers find these through their day-to-day engagement with the platform. The illusion of control can operate relatively effectively in the regular operation of the platform, but suffers when workers struggle against control (Woodcock, 2020). For example, during wildcat strikes which have become a frequent form of protest on platforms (Joyce et al., 2020), there a few options left to the platform, other than introducing boosts to the piece rate.

## Struggles over technology

One of the important things that is missing from the panopticon metaphor, either in the call center or with platforms, is that it tends to hide the planner of the system. Artificial intelligence is not neutral and is instead designed for particular purposes. As with the automation of factories, the choices made about the kinds of technologies used and how they are implemented is about more than just efficiencies at work (Noble, 1978).

There are many examples of ways in which workers have circumvented algorithmic control in practice in platform work (Woodcock, 2021), but we know less about the choices that happen inside these companies to implement the technology. However, as Braverman (1998, p. 137) reminds us, capital became built into the machinery of factories:

Thus, as the process takes shape in the minds of engineers, the labor configuration to operate it takes shape simultaneously in the minds of its designers, and in part shapes the design itself. The equipment is made to be operated; operating costs involve, apart from the cost of the machine itself, the hourly costs of labor, and this is part of the calculation involved in machine design. The design which will enable the operation to be broken down among cheaper operators is the design which is sought be management and engineers who have so internalized this value that it appears to them to have the force of natural law or scientific necessity.

Historically, the introduction of machines has been part of a concerted attempt to undermine workers power in the workplace. For example, “machinery offers to management the opportunity to do by wholly mechanical means that which it had previously attempted to do by organizational and disciplinary means” (Braverman, 1998, p. 134). Machines provided the opportunity to set and control the pace of work centrally, shifting the balance of power away from workers. The application of technology is not only about efficiency, but also as an attempt at control.

In order to understand the implications of artificial intelligence at work, any analysis needs to consider how this new application of technology builds upon previous interventions in the labor process over a long history of struggles at work. First, artificial intelligence needs to be interrogated, rather than taken for granted. Research needs to critically unpack the relationships involved in the development, use, and resistance to new applications. Second, there are a wide range of forms that artificial intelligence can take. If they are involved in controlling—or attempting to control—work, these can be unpacked further by considering what role they play within the control of the labor process: direction, evaluation, and/or discipline. No system of control at work can operate without bringing these components together and they often rely on human manager/supervision intervention at some point within or across these. This involves understanding how data collection, no matter how complex the data are or how rapidly it can be achieved, is only one part of the process. Data needs to be acted on to become and attempt at control. Third, arguments about artificial intelligence at work need to be put into conversation with the theoretically and empirically rich traditions of labor process theory.

Future research is needed on how specific applications of artificial intelligence are operating in practice in different kinds of work. As the examples of the call center and transport platforms show, the reality of using technology within the labor process is far from straightforward. Empirical studies provide an important way to move our understanding of the implications of different kinds of artificial intelligence at work forward, particularly moving beyond the claims or marketing that are associated with them. Rather than general research, what is needed is critical research that searches for the contradictions, conflicts, and struggles along the supply chains of artificial intelligence. This is part of situating artificial intelligence as a technology that emerges from, and is used within, the existing social relations at work and in society.

Future research can also benefit from analyzing the different types of struggles against power. For example, Foucault notes that there can be struggles “either against forms of domination; against forms of exploitation which separate individuals from what they produce; or against that which ties the individual to himself and submits him to others in this way” (Foucault, 1982, p. 781). Understanding struggles against artificial intelligence at work can be understood through these different types. Is a struggle aimed at domination, exploitation, or against forms of subjectivity and submission more widely? For example, Moore (2022) recent research on data subjects points toward this with emerging struggles for subjectivity. While some may herald artificial intelligence as driving change within the contemporary world, attention needs to be drawn to the interests it serves and the relationships of power, as well as how other interests can struggle against this too.

## Data availability statement

The original contributions presented in the study are included in the article/supplementary material, further inquiries can be directed to the corresponding author.

## Author contributions

The author confirms being the sole contributor of this work and has approved it for publication.

## Conflict of interest

The author declares that the research was conducted in the absence of any commercial or financial relationships that could be construed as a potential conflict of interest.

## Publisher's note

All claims expressed in this article are solely those of the authors and do not necessarily represent those of their affiliated organizations, or those of the publisher, the editors and the reviewers. Any product that may be evaluated in this article, or claim that may be made by its manufacturer, is not guaranteed or endorsed by the publisher.
